# Linking bronchopulmonary dysplasia to adult chronic lung diseases: role of WNT signaling

**DOI:** 10.1186/s40348-016-0062-6

**Published:** 2016-10-07

**Authors:** Chiharu Ota, Hoeke A. Baarsma, Darcy E. Wagner, Anne Hilgendorff, Melanie Königshoff

**Affiliations:** 1 Comprehensive Pneumology Center, Helmholtz Center Munich, Ludwig-Maximilians-University, University Hospital Grosshadern, German Center of Lung Research (DZL), Munich, Germany; 2 The Perinatal Center, Campus Grosshadern, Ludwig-Maximilians-University, Munich, Germany

**Keywords:** Bronchopulmonary dysplasia (BPD), WNT signaling, Lung development, Adult chronic lung diseases

## Abstract

Bronchopulmonary dysplasia (BPD) is one of the most common chronic lung diseases in infants caused by pre- and/or postnatal lung injury. BPD is characterized by arrested alveolarization and vascularization due to extracellular matrix remodeling, inflammation, and impaired growth factor signaling. WNT signaling is a critical pathway for normal lung development, and its altered signaling has been shown to be involved in the onset and progression of incurable chronic lung diseases in adulthood, such as chronic obstructive pulmonary disease (COPD) or idiopathic pulmonary fibrosis (IPF). In this review, we summarize the impact of WNT signaling on different stages of lung development and its potential contribution to developmental lung diseases, especially BPD, and chronic lung diseases in adulthood.

## Introduction

Bronchopulmonary dysplasia (BPD) is one of the most common chronic lung diseases in infants. “Old” or “classical” BPD was first defined by Northway et al. in 1967 as structural lung damage and subsequent appearance of parenchymal fibrosis caused by prolonged hyperoxia and ventilator-associated lung injury during the saccular to alveolar stage of lung development [1]. Improvement of clinical neonatal intensive care practices, including prenatal steroid therapy, exogenous surfactant administration, protective lung ventilation strategies, and the careful monitoring of oxygen supplementation, has led to a significant reduction in perinatal respiratory-associated death. With the current clinical practices, newborns as early as 23 to 26 weeks of gestation are able to survive; however, these newborns present with a distinct form of “new” BPD. The prominent new BPD comprises arrested alveolarization and vascularization, due to the impact of different risk factors on the functionally and structurally immature lung during the early canalicular and saccular periods of lung development [2]. Risk factors include hyperoxia-induced oxygen toxicity, mechanical ventilation-induced lung injury, and infection/inflammation of the lungs, which results in aberrant lung development due to extensive remodeling of the extracellular matrix (ECM), perturbations of inflammatory response, and impaired growth factor signaling [3]. Newborns which have survived and developed new BPD are approaching adolescence and adulthood. Several longitudinal studies following patients with new BPD have demonstrated a decline of forced expiratory volume in 1 second (FEV1) compared with term-born controls, reflecting the development of airflow obstruction in new BPD survivors over time [2, 4–6]. These data indicate that impairment of alveolarization/vascularization during childhood, which is a feature of the new BPD, might contribute to deranged lung alveolar injury/repair processes in adulthood.

Environmental insults, such as smoking, infection, or hyperoxia, are known to cause aberrant alveolar repair processes in deranged lung development and also the adult lung [7, 8]. These environmental insults contribute not only BPD but also other childhood respiratory diseases including bronchial asthma. A better understanding of the processes and signaling pathways altered by these insults are clearly needed.

Impaired signaling of essential lung development pathways, such as fibroblast growth factor (FGF) [9, 10], Wingless/integrase-1 (WNT) signaling [11], or bone morphogenetic proteins (BMPs) [12], have been reported to contribute to the pathogenesis of adult chronic lung diseases, such as chronic obstructive pulmonary disease (COPD) or idiopathic pulmonary fibrosis (IPF) [13]. Of particular interest, WNT signaling has been linked to aberrant alveolar epithelial injury and repair processes [11, 14–17]. Because these pathways are mostly attributed to lung development and are normally thought to be quiescent in the adult lung, this raises the question of why these pathways become aberrant in the adult and whether pre- or postnatal insults impact developmental signal activity early on, thus contributing to an increased susceptibility for chronic lung diseases later in life [7, 18]. In this review, we focus on the potential role of WNT signaling in lung development and perinatal lung disease, with a focus on BPD, as a disease of impaired alveolarization/vascularization, and discuss the potential link between perinatal and adult chronic lung diseases, such as COPD and IPF.

## Review

### Overview of WNT signaling

**Fig. 1:**
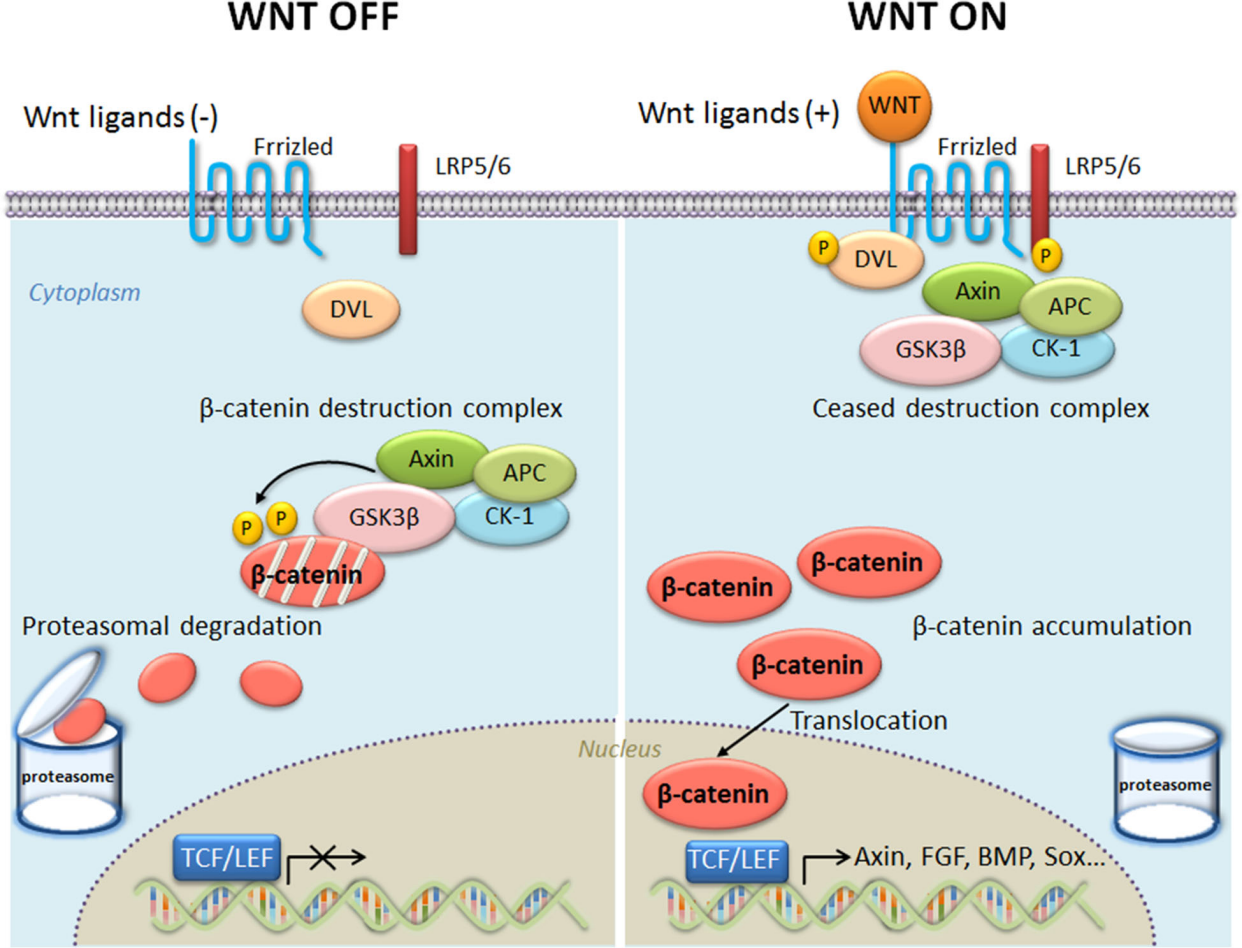
Overview of WNT/β-catenin signaling. Without WNT signaling (“WNT OFF”), “destruction complex phosphorylates cytosolic β-catenin and phosphorylated β-catenin is recognized and degraded by the proteasomes. With WNT signaling (“WNT ON”), the function of “destruction complex” is inhibited to phosphorylate cytosolic β-catenin. Then unphosphorylated β-catenin accumulates in the cytosol, translocates into the nucleus, and activates the WNT target gene expression, such as the T-cell factor and lymphoid enhancer factor-1 (TCF/LEF1) family of transcription factors

WNT signaling is a critical pathway for embryonic development and adult cellular injury and repair processes. There are at least three well-known WNT pathways: canonical (β-catenin dependent) signaling and two non-canonical pathways, (i) planar cell polarity (PCP) and (ii) Ca^2+^-calmodulin-dependent protein kinase II (Camk II)/protein kinase C (PKC) signaling. As shown in Fig. 1, canonical WNT/β-catenin signaling mainly consists of; (i) WNT ligands, (ii) the transmembrane receptors, Frizzled (FZD_1–10_), (iii) the co-receptors low-density lipoprotein receptor-related proteins (LRP) 5 and 6, (iv) signaling intermediates, Dishevelleds (DVL1–3), (v) the β-catenin “destruction complex”, (vi) the transcriptional co-activator, β-catenin, and (vii) the transcription factors, T cell factor and lymphoid enhancer factor (TCF/LEF). Extracellular modulators, such as Dickkopfs (DKK1–4), WNT-inhibitory factor-1 (WIF1), or secreted Frizzled-related proteins (SFRPs), are also important for regulation of the pathway. In the absence of WNT ligands, β-catenin is phosphorylated by the destruction complex, which is comprised of Axin, adenomatous polyposis coli (APC), glycogen synthase kinase-3 beta (GSK-3β), and casein kinase-1 (CK1). Phosphorylated β-catenin is recognized and ubiquitinated by ubiquitin ligase E3 and subsequently degraded by the proteasome. Upon WNT ligand binding to its receptors, the capacity of the destruction complex to phosphorylate cytosolic β-catenin is inhibited. Unphosphorylated β-catenin accumulates in the cytosol, translocates into the nucleus, and activates WNT target gene expression, via its integration with the TCF/LEF family of transcription factors, which is important for cellular proliferation, differentiation, and survival [11, 19, 20].

Non-canonical WNT signaling (i.e., β-catenin independent) mainly consists of (i) the WNT/PCP pathway, which activates c-Jun-N-terminal kinase (JNK) and proteins associated with cytoskeleton rearrangement, and (ii) the WNT/Ca^2+^ pathway, activating Camk II, PKC, the transcription factor nuclear factor of activated T cells (NFAT), and several other (less well defined) transcription factors [19]. In this review, we primarily focus on the canonical WNT/β-catenin signaling, which has been investigated most extensively so far.

### Lung development and WNT signaling

**Fig. 2:**
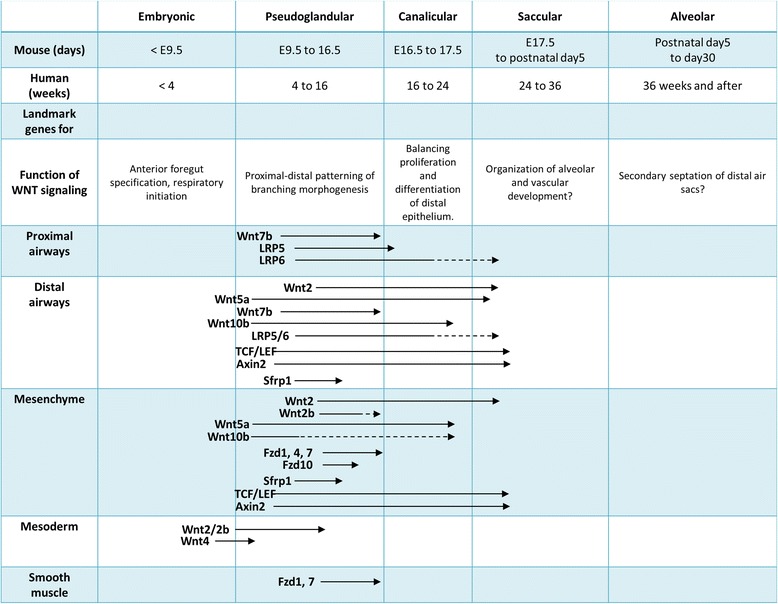
Developmental stages and the WNT/β-catenin signaling. In the embryonic stage (<E9.5 in mouse, <4 weeks in human), Wnt2/2b is expressed within surrounding mesenchyme for the anterior foregut specification and the onset of lung specification. In the pseudoglandular stage (E9.5 to 16.5 in mouse, 4 to 16 weeks in human), several Wnt ligands, receptors, and extracellular modulators are expressed within proximal or distal airways and surrounding mesenchyme and coordinate proximal-distal patterning of branching morphogenesis. In the canalicular stage (E16.5 to 17.5 in mouse, 16 to 24 weeks in human), WNT/β-catenin signaling regulates the balance of proliferation and differentiation of the distal epithelium. In the saccular to alveolar stage (E17.5 to postnatal day 5, postnatal day 5 to day 30 in mouse, 24 weeks to 36 weeks, 36 weeks and after in human), WNT/β-catenin signaling may organize the alveolarization and vascularization and the formation of secondary septa

Historically, a large portion of our knowledge about lung development has been obtained by using wild-type or transgenic mice [21]. In the mouse lung, embryonic lung development starts as early as E9.5 (equivalent to 4 weeks in human gestation), with tightly coordinated epithelial and mesenchymal differentiation processes, and is completed postnatally. At this time point, *Nkx2.1*, a critical homeodomain-containing transcription factor for initial respiratory specification, is expressed within endoderm progenitors in the anterior foregut [21]. Dorsal-ventral specification occurs according to signals, such as BMPs, FGFs, or WNTs, from the surrounding mesenchyme, endoderm, or mesoderm. Primary lung buds generate tree-like structures for branching morphogenesis from E9.5 to E16.5 (in human, 4 to 16 weeks, historically called the “pseudoglandular stage”), followed by the “canalicular stage” (E16.5–17.5 in mouse, 16 to 24 weeks in humans) when terminal sacs are formed, the “saccular stage” (E17.5 to postnatal day 5 in mouse, 24 to 36 weeks in human) when distal airways are developed for the alveoli, and the “alveolar stage” (postnatal day 5 to 30 in mouse, 36 weeks and after delivery in human) when secondary alveolar septa are formed to further divide the airspaces into definitive alveoli (Fig. 2).

WNT signaling is active and highly controlled in a spatio-temporal fashion throughout murine lung endoderm specification in the foregut as well as cellular proliferation and differentiation during lung development [21–24]. In the mouse lung, several loss- or gain-of-function studies revealed the importance of WNT signaling in lung morphogenesis [25, 26]. Here, we review the role of active WNT signaling during murine lung development (summarized in Table 1).

**Table 1:** Summary of developmental WNT/β-catenin signaling, expression pattern, study models, and phenotypes

	Expression	Study model	Phenotype	Reference
WNT ligands				
WNT2	MS	*Wnt2* knockout	1. Hypoplastic lungs with relatively normal airway development 2. Reduced proliferation of EP and MS lineage 3. Several signaling pathways and transcription factors for lung development were reduced	[27]
WNT2b	MS	*Wnt2b* knockout	Viable and no discernable phenotype	[27]
WNT2/2b	MS	*Wnt2/2b* double knockout	Complete lung agencies	[27]
WNT4	MS	*Wnt4* knockout	1. Lung hypoplasia and tracheal abnormalities 2. Reduced mesodermal proliferation in the lung bud	[30]
WNT5a	MS, EP(E12) Distal and proximal EP (E16) Cells surrounding distal and proximal EP (E18)	*Wnt5a* knockout *Wnt5a-*SPC transgenic	1. Larger lungs, foreshortened trachea, overexpansion of distal airways, thickened intersaccular interstituim (knockout) 2. Smaller lungs, reduced number of alveolar sacs with dilated alveoli, lobation abnormalities (transgenic)	[31] [32]
WNT7b	EP (E12.5 to E16.5) both in the distal and larger mainstem bronchial airways	Conditional knockout of *Wnt7b* in *Sox2*-expressed embryo	1. Hypoplastic lungs with normal patterning and cell differentiation 2. Proportionate decrease in the replication of epithelial and mesenchymal progenitors	[29]
WNT receptors				
FZDI	MS	In situ hybridization	NA	[33]
FZD2	EP (distal)	Conditional knockout of *Frz2* in Shh-expressing cells	Formation of cysts in distal airways and defective branching morphogenesis	[35, 36]
FZD4	MS	In situ hybridization	NA	[33]
FZD7	MS	In situ hybridization	NA	[33]
FZD8	EP	In situ hybridization		[34]
FZD10	EP (distal)	Immunohistochemistry	NA	[33]
LRP5	EP (upper airway), muscular component of large vessels	*Lrp5* knockout	Impairment of alveolar and vascular formation in neonatal lungs due to the decrease of angiopoietin/Tie2 pathway	[33, 37]
LRP6	EP (upper airway)	In situ hybridization	NA	[33]
Extracellular modulators				
DKK-1	EP (distal)	In situ hybridization	Treatment of E11.5 lung explants by Dkk1 disrupts branching morphogenesis	[34]
SFRP-1	MS, EP (distal)	*Sfrp-1* knockout	Marked dilation of the alveolar duct with the loss of surrounding messenchymal component	[43]
β-catenin destruction complex				
APC	MS	Conditional knockout of *Apc* in *Tbx4-*expressing mesenchymal cells	APC knockout fetus shows severe lung hemorrhage in E14.5 and dies in E15.5, with condensed mesenchymal cells around epithelial tubes in the lung.	[44, 45]
β-catenin				
β-catenin	EP, MS	1. Conditional knockout of β-catenin in SPC-expressing cells 2. Conditional knockout of β-catenin in Shh-expressing cells 3. Conditional knockout of β-catenin in Sox2-expressing cells 4. Conditional knockout of β-catenin in Demol-expressing mesenchymal cells	1. Multiple, enlarged, and elongated bronchiolar tubes with a lack of alveolar sacs (β-catenin-SPC knockout) 2. Absence of both trachea and lung due to the defect of Nkx2.1 expression (B-catenin-Shh knockout) 3. Defective bronchiolar epithelial cell differentiation and marked ectasis of the developing and adult airway (β-catenin-Sox2 knockout) 4. Shortened trachea and reduced branching morphogenesis. Defect of sub-mesothelial mesenchymal domain containing Fgf10-expressing progenitors.	[26, 28, 38–40]

*MS* mesenchymal cells, *EP* epithelial cells

### WNT ligands and their receptors in the developing mouse lung

A number of WNT ligands and receptors have been identified as being critical for various stages of development. Deletion of the canonical *Wnt2* ligand causes mouse lung hypoplasia whereas *Wnt2/2b* double knockout leads to complete lung agenesis in mice with a loss of *Nkx2.1* in early embryonic development in the region where the lung buds are derived from the foregut. Thus *Wnt2* and *2b* are required to specify the *Nkx2.1*-expressed lung progenitors in the foregut through canonical WNT/β-catenin signaling [27, 28]. Similarly, deletion of murine *Wnt7b* results in hypoplastic lungs with a proportionate decrease in the replication of both epithelial and mesenchymal progenitors [29]. The non-canonical *Wnt4* was reported to be expressed in the anterior trunk mesoderm and was found to be essential for proper lung morphogenesis and trachea formation. In *Wnt4* knockout mice, reduced mesodermal proliferation in the lung bud leads to severe lung hypoplasia and tracheal abnormalities [30]. Moreover, *Wnt5a*, another ligand of non-canonical WNT signaling, has been detected as early as E12 at both epithelial and mesenchymal compartment of the developing lung. The absence of *Wnt5a* activity is associated with the overbranching of distal airways in murine E15–16 lung together with an architectural immaturity of the capillaries and alveolar airspaces [31]. Vice versa, *Wnt5a* overexpression in the distal epithelium results in reduced epithelial branching and dilated distal airways [32]. These data highlight that both canonical as well as non-canonical signal aberrations affect normal lung development.

In addition to WNT ligands, the receptors have also been shown to be important for proper lung development. Tissue-specific analysis of the WNT receptors from E12.5 to E16.5 revealed FZD1, 4, and 7 to be primarily expressed in the developing mouse lung mesenchyme and FZD10 in distal airway epithelium and the expression of those receptors decreases after E14.5 [33]. FZD2 is also highly expressed in distal airways [34], and epithelium-specific deletion of *Fzd2* causes formation of cysts in distal airways and defective branching morphogenesis [35]. Furthermore, FZD8 is expressed throughout the pulmonary epithelium [36]. Both LRP5 and LRP6 are expressed in the upper airway epithelium, and LRP5, but not LRP6, is also expressed in the smooth muscle compartment of large vessels [33]. Loss of *Lrp5* inhibits angiogenesis and alveolar formation in neonatal mice in the alveolar stage of lung development [37]. While these data further underline the importance of WNT signaling during development, much less is known how these receptors are distinctly involved in canonical versus non-canonical WNT signaling. Furthermore, future studies elucidating cell-specific expression under (patho-) physiologic conditions are needed.

### β-Catenin in normal lung development

Studies modulating the major effector protein of canonical WNT signaling, β-catenin, revealed that both epithelial and mesenchymal β-catenin is required for the onset of lung specification and the proximal-distal patterning of branching morphogenesis: deletion of β-catenin in the foregut leads to the loss of *Nkx2.*1 expression and the absence of both the trachea and lung due to a lack of respiratory lineage initiation [27, 38]. Lung epithelium-specific deletion of β-catenin caused disruption of distal but not proximal airways in the later stages of lung development [28]. On the other hand, conditional knockout of β-catenin in mesenchymal cells resulted in a shortened trachea and reduced branching morphogenesis in E12.5 to E14.5 lungs [23]. In addition to the localization, the timing and duration of β-catenin expression are important for proper lung development. Prolonged activation of a β-catenin-Lef1 fusion protein in distal lung endoderm led to the development of an undifferentiated distal airway epithelium and resulted in ectopic expression of gene characteristic of intestinal epithelial lineages [26]. Another study revealed that sustained β-catenin activity within the distal lung endoderm in early lung development results in the loss of *Sox2*, a regulator of developing proximal airway progenitors, and defective bronchiolar epithelial cell differentiation and marked ectasia of the developing and adult airway [39]. β-Catenin appears to be an essential mediator with critical links to its target transcription factors as well as various signaling pathways associated with lung development, such as, FGF [40], BMP4, or N-myc [41] signaling.

#### WNT signal antagonists in the developing mouse lung

Activation of WNT signaling is highly controlled by several intra- and extracellular proteins. Dickkopf (DKK1-4) proteins antagonize WNT/β-catenin signaling by binding to LRP5/6. *Dkk-1*, *2*, and *3* are expressed in distal lung epithelium from E11.5 (*Dkk-2*), E12.5 (*Dkk-3*), and E13.5 (*Dkk-1*) onward [36]. WNT/β-catenin signaling was decreased in distal airways of TOPGAL WNT-reporter mouse at E14.5 after the onset of *Dkk-1* expression in the distal lung [36]. Furthermore, the DKK-1-treated lung explants from E11.5 mice showed impaired branching, failed cleft formation, and enlarged terminal buds with fibronectin deposition [36]. Retinoic acid (RA) is a known canonical WNT activator. Using mice with an RA-deficient lung foregut, it was shown that WNT/β-catenin signal activation, at the onset of lung specification, is dependent on the repression of *Dkk-1* by endogenous RA [42], indicating that repression and expression of DKKs takes part in WNT/β-catenin signaling coordination during lung development.

SFRP-1, which antagonizes WNT/β-catenin signaling by binding extracellular WNT ligands, can also be detected in the distal epithelium and the surrounding mesenchyme from E13.5 to 15.5; however, it has not been observed in the later stages of lung development [43]. In *Sfrp-1* knockout mice, it was shown that nuclear β-catenin levels were enhanced after E16.5 to E18.5 and that the alveolar ducts were dilated postnatally compared with the wild-type mice [43], indicating that SFRP-1 is involved in the coordination of nuclear β-catenin translocation for proper alveolarization.

APC, a component of the intracellular β-catenin destruction complex, is highly expressed in mesenchymal cells surrounding the large airways at E14.5 lung and can be detected in both mesenchymal as well as epithelial cells at E18.5 [44]. Lung mesenchyme-specific conditional *Apc* knockdown results in hyperactivation of β-catenin in embryonic lung mesenchyme at E10.5 and fetal death at E15.5 due to massive pulmonary hemorrhage. Histological analysis of *Apc* knockout mice revealed abnormal proliferation and disrupted differentiation of pulmonary mesenchymal cells and inhibition of branching morphogenesis and vasculogenesis [45].

Taken together, WNT signal components, including several antagonists, are involved in a variety of critical processes during murine lung development, including the onset of lung specification, branching morphogenesis, alveolar formation, and angiogenesis with tightly coordinated epithelial and mesenchymal expression patterns for each time point and developmental stage. As such, it is reasonable to speculate that perturbation of WNT signaling on several levels during lung development may lead to the arrest of alveolarization and vascularization as observed clinically in BPD.

In contrast to mouse lung development, our knowledge about WNT signaling in human lung development remains sparse. To date, only two studies exist that reports on WNT signaling expression in developing human lung. Zhang et al. performed quantitative PCR and in situ hybridization using a developing human lung and showed that messenger RNA (mRNA) expression of *WNT2*, *WNT7B*, *FZD4*, *FZD7*, *LRP5*, and *LRP6* was restricted to the alveolar and bronchial epithelium in the human lungs at 7, 12, 17, and 21 weeks of gestation [46]. Most WNT components were up-regulated gradually until 17 weeks and subsequently decreased in 21 weeks of gestation [46]. Sharma et al. performed differential gene expression analysis using human lung tissue samples across pseudoglandular and canalicular stages of development and DNA samples obtained from two cohorts of childhood bronchial asthma [47]. They showed that both WIF1 and Wnt1-inducible signaling pathway protein-1 (WISP1) are associated with intrauterine airway development and lung function impairment in childhood asthmatic patients [47].

Notably, there are several differences between mouse and human lung development in terms of cellular composition, timing of branching morphogenesis, or alveolar maturation [21, 48]. In the mouse lung, a pseudostratified epithelial layer including basal cells are only found in the trachea and main stem bronchus, whereas it is extended into terminal bronchioles in the human lung. In addition, club cells are found throughout mouse airways, while they are only found in the bronchiolar epithelium in the human lung. The number of the branches in the bronchial tree is higher in humans compared to mouse lungs. The lobation or branch pattern also differs between human and mouse [49]. Alveolar formation started from saccular stage, E17.5, in mouse, and around 24 weeks, late canalicular stage in human [48]. Considering these differences, further approaches to decipher the role of developmental signaling pathways in human lung development are required, using, e.g., human histological samples [50] or human induced pluripotent stem (iPS) cells [48].

### Involvement of WNT signaling in early lung injury and adult chronic lung diseases

A number of environmental insults during pre- and postnatal development are known to induce deranged lung morphogenesis and have also been shown to affect WNT signaling. Several studies have been conducted to investigate whether insults to the developing lung by incidental environmental factors, such as postnatal infection or maternal cigarette smoke as well as medical interventions, such as ventilation and oxygen supplementation, affect lung morphogenesis and repair process via WNT signaling. Neonatal hyperoxia is one of the widely used animal (mainly rodent) models to mimic BPD, i.e., causing impaired alveolarization/vascularization in neonatal lungs [51]. Neonatal hyperoxia following maternal bacterial infection is another rodent model for BPD [51, 52]. Since BPD is caused by a variety of factor, including hyperoxia exposure, intrauterine infection, high-pressure ventilation, or prematurity of the lungs, rodent models of BPD, including hyperoxia exposure, do not completely recapitulate the BPD observed in clinical settings. However, because the lung samples from human neonates are rare, those rodent models of BPD, including hyperoxia exposure models focusing especially on alveolarization/vascularization, are important tools to reveal the pathophysiology of new BPD.

These pre- and postnatal insults can result in a variety of early lung diseases next to BPD. In particular, bronchial asthma exhibits a high incidence in childhood and adolescence. Bronchial asthma is considered to be highly influenced by maternal smoking [53], diet [54], intrauterine growth restriction [55, 56], or exposure to pathogens [57, 58]. In this next section, we discuss the environmental insult-associated with altered WNT/β-catenin signaling in lung injury occurring during early lung development and its potential contribution to adult chronic lung diseases.

#### Infection and inflammation

Prenatal infections are known to impact lung development. Premature rupture of the amniotic membrane results in increased susceptibility to intrauterine infections. Antenatal inflammation of chorioamniotic membranes causes premature birth and adversely affects lung development [59]. Intra-amniotic lipopolysaccharide (LPS) exposure, which mimics amniotic bacterial infection, decreases the expression of *Lef-1*, *Wnt1*, *Wnt4*, and β-catenin in the canalicular stage of lung development [60]. Similarly, in adult mice, acute lung injury caused by intra-tracheal application of LPS and followed by high tidal volume mechanical ventilation results in the activation of DKK1 and the subsequent down-regulation of active β-catenin in the lung alveolar epithelium. It was shown that DKK-1 is released from activated platelets, and the binding affinity of DKK-1 to alveolar epithelial cells was increased during acute lung inflammation [61]. Although only a few reports have shown the relationship between early lung infection/inflammation and WNT signaling so far, the data to date are intriguing and further studies investigating human lungs undergoing prenatal infections, such as chorioamnionitis-induced neonatal lung injury, will be important.

#### Smoking-related injury

Maternal smoking is one of the important risk factors for chronic lung diseases in children including recurrent respiratory infection, infantile wheezing, bronchial asthma, and lower respiratory function in early adulthood [53, 62, 63]. It has also been shown that maternal smoking affects alveolarization/vascularization in developing lung in vivo [64, 65]. Bronchial asthma shares some similarities with BPD (e.g., pathologic airways and the presence of clinical symptoms like wheezing) and has been associated with aberrant WNT signaling as well [47, 66]. Impaired lung growth by these and other environmental factors may cause the formation of smaller airways and decreased lung capacity contributing to childhood asthma and lower respiratory function in early adulthood [67]. In the adult lung, dysfunction of WNT signaling contributes to the impaired epithelial repair processes in disease [17, 68]. WNT signaling is reduced in the lungs from COPD patients, a smoking-related disease, and the pharmacological activation of the signaling pathway through GSK3β inhibition activates epithelial repair properties and attenuates known pathological features of emphysema ex vivo [68] and in vivo [17, 69]. Recently, Jiang et al. reported that FAM13A, a gene associated with COPD susceptibility [69], might lead to emphysema development by facilitating β-catenin degradation [70]. Furthermore, WNT/β-catenin signaling components, including canonical WNT ligands, FZDs, signal transducers, and target genes are down-regulated, while antagonists such as SFRP-1 and DKK-1 are up-regulated, in human lung tissue [43] and in particular in the small airway epithelium [71, 72] from COPD patients.

Much less is known about the effects of maternal smoking on WNT signaling during lung development. Maternal smoking during pregnancy has been shown to decrease *Fzd7* and *Ctnnb1* (β-catenin) mRNA in neonatal Balb/c mice [73]. Furthermore, it was recently reported that protein and mRNA expression of *SFRP-1* were significantly up-regulated in the placental tissues in smoking women compared with those from non-smokers [74]. Furthermore, a carbon monoxide analog, one of the components in cigarettes, increased *SFRP-1* expression accompanied by decreased WNT/β-catenin signaling in a human trophoblast cell line. In addition, maternal *Sfrp-1* overexpression causes fetal growth restriction in mice [74]. Altogether, these studies strongly suggest that (maternal) smoking and components of cigarette smoke significantly impact WNT signaling activity. However, cigarette smoking is also known to generally inhibit fetal growth and thus it remains an open question whether maternal smoking/nicotine exposure directly increases extracellular modulators of WNT and decreases WNT/β-catenin signaling in the lung to affect fetal lung development or whether maternal smoking/nicotine exposure induces fetal growth restriction to cause premature birth and subsequent BPD.

Given these studies, it is plausible that chronic lung diseases, such as COPD, develop as a result of early lung insults leading to aberrant WNT/β-catenin signaling and thus lung repair capacity. A gradual decline in lung function in early adulthood might be due to aberrant WNT/β-catenin signaling, which is retained over time, eventually resulting in adult chronic lung diseases. Generation of experimental models to follow this hypothesis and long-term follow-up studies of new BPD patients are needed. In particular, emerging evidences suggest that epigenetic alterations, i.e., modified gene expression via DNA methylation, histone modification, or microRNA, of WNT signaling represents an important area of investigation. Recently, it has been shown that cigarette smoke exposure epigenetically altered WNT/β-catenin signaling in lung cancer cells by histone modification or microRNA expression [75–77]. In the developing lung, differential methylation of WNT/β-catenin signal genes have been reported in neonatal and adult mouse lungs [78]. Although there is no study addressing epigenetic alterations of WNT signaling by cigarette smoke exposure in the developing lung, it is reported that psychological stress during pregnancy caused altered DNA methylation of non-canonical, WNT5a/Ca^2+^ pathway and postnatal wheeze of the affected children [66]. As such, further investigations on how environmental factors, including maternal smoking, alter WNT signaling by epigenetic modifications and thus affect lung development of the neonate will be important.

#### WNT/β-catenin and TGF-β signaling in new BPD

In addition to WNT/β-catenin, transforming growth factor (TGF)-β signaling is a critical pathway for lung development [21]. It has been shown that TGF-β is an important mediator for the development of BPD [79–81] and is activated in neonatal rat lungs after hyperoxia exposure [82] as well as in neonatal mouse lungs after mechanical ventilation with mild hyperoxia [83]. There are also reports regarding the dual activation of WNT/β-catenin and TGF-β signaling in hyperoxia exposure models, but the crosstalk between the two pathways is incompletely understood [82, 84]. Active WNT/β-catenin signaling has been reported in fibrotic adult lung diseases, such as IPF [14–16], in which TGF-β signaling is highly involved in epithelial cell reprogramming and myofibroblast activation [85]. Furthermore, TGF-β results in enhanced expression of WNT ligands and activation of β-catenin in vitro [86]. TGF-β-induced activation of WNT/β-catenin signaling [87, 88] may also play a key role during developing BPD as well as adult fibrotic lung diseases, including IPF.

#### WNT signaling in BPD

To date, only a few studies addressed WNT signaling in new BPD patients. In the lungs of patients who died from BPD, nuclear β-catenin, which is used as a surrogate marker for WNT/β-catenin activity, along with phosphorylated (inactivated) GSK-3β was found in the thickened alveolar septa [89, 90]. Notably, whole exome sequencing using blood spots from twin neonate pairs with and without BPD revealed that genes associated with WNT/β-catenin signaling were up-regulated in BPD [91].

Nuclear translocation of β-catenin and increased Lef1 expression was observed in the lung from neonatal rats exposed to hyperoxia (95 % oxygen) in the alveolar stage (postnatal days 0 to 7) [82]. Moreover, it has been shown that neonatal hyperoxia increased nuclear β-catenin and decreased alveolar epithelial type II (ATII) cell to ATI cell transdifferentiation [84, 92], which is generally considered as a repair process of alveolar epithelial cells following injury. Furthermore, hyperoxia-induced inhibition of ATII to ATI transdifferentiation was recovered by small interfering RNA (siRNA)-mediated knockdown of Wnt3a in vitro [92]. However, it has been reported that in the adult mouse lung, β-catenin was induced during ATII cell to ATI cell transdifferentiation in normoxia condition [93, 94]. This discrepancy might be due to hyperoxia condition or using neonatal lung in the former studies. Further studies are needed to clarify this issue. Another study showed the enhancement of WNT/β-catenin signaling in impaired vascularization [95]. Taken together, canonical WNT/β-catenin signaling is activated in lung samples from BPD patients and neonatal rodent model of hyperoxia exposure in the lung. This activation of WNT signaling might be a result of “attempted (and failed)” regeneration after injury of alveolar epithelial cells, which is a hypothesized processes model in IPF lungs [11].

It is unclear whether BPD contributes to the onset of adult chronic lung diseases, such as COPD or IPF. In adult chronic lung diseases, canonical WNT/β-catenin signaling is down-regulated in emphysematous lungs [17] while up-regulated in fibrotic lungs [15]. In BPD lungs, as mentioned above, enhanced expression of TGF-β [79] and/or WNT/β-catenin signaling was observed [89, 90]. In contrast, intrauterine infection or cigarette smoke decreased WNT/β-catenin signaling. Longitudinal studies showed that along with decreased FEV1, a hallmark of obstructive lung diseases, forced vital capacity (FVC), a hallmark of restrictive lung diseases, was also lower in BPD survivors [96, 97]. Although characterizing a disease entity as either emphysema or fibrosis is oversimplification, it seems like BPD features a co-existence of emphysema and fibrosis as reported [98, 99]. It is possible that different environmental insults at different time points during lung development might cause different expression patterns of WNT signaling. Also, if new BPD survivors with impaired alveolarization/vascularization are exposed to a “second hit,” such as cigarette smoke, pathogens, or hyperoxia, at a later time point, they might develop adult chronic lung diseases with aberrant (increased/decreased) WNT signaling. Longitudinal studies are needed to study whether BPD survivors are more susceptible to developing adult chronic lung diseases. Establishing animal models to mimic BPD and follow the outcome of the developing lung is needed.

### Clinical implications and limitations of WNT/β-catenin signaling in lung development

Several studies indicated that targeting WNT/β-catenin signaling may be a therapeutic strategy in BPD. Vitamin A, whose metabolite is RA, has been used to prevent BPD progression [100–102] although the effect is still controversial. As mentioned previously, RA activated WNT/β-catenin signaling via inhibition of DKK-1 [42]. In this context, active WNT/β-catenin signaling might be beneficial for arrested alveolarization, as it has been reported to maintain alveolar stem/progenitor cell populations [11, 103], such as ATII cells [94].

On the other hand, it is reported that intraperitoneal administration of Mesd, a specialized chaperone for LRP5/6 to inhibit WNT/β-catenin signaling, attenuated hyperoxia-induced pulmonary hypertension and right ventricular hypertrophy in neonatal rats [95]. Another study showed that ICG-001, a small molecule which inhibits WNT/β-catenin signaling via interaction between β-catenin and CREB-binding protein (CBP), an intrinsic histone acetyltransferase to activate gene transcription, increased alveolarization and decreased vascular remodeling to develop pulmonary hypertension [104]. Resveratrol, a polyphenol found in several fruits and nuts, was also shown to attenuate hyperoxia-induced model of BPD in neonatal rats [64, 84]. It is important to address the question when or where WNT/β-catenin signaling should be inactivated/activated for physiologic lung development and perinatal lung injury/repair processes. Thus, it is worth exploring whether the attenuation of alveolar repair process or epigenetic modification altering WNT signaling will be candidates for clinical implications.

However, as most of the data shown here originate from rodent experiments, limitations for translation of the findings have to be considered. Given the difficulty of obtaining human neonatal lung tissue for analysis, recent approaches using 3D lung tissue cultures [68] or iPS cells [105, 106] from BPD patients represent promising tools to further explore signaling pathways involved in the pathogenesis of disease, such as WNT/β-catenin signaling. Collecting more evidence from preterm infants will be needed to identify new therapeutic targets in WNT/β-catenin signaling pathway.

## Conclusions

Here, we discussed the potential role of the developmental WNT signaling pathway as a potential missing link between early impairment of lung development and the outcome in the adult lung. First, WNT/β-catenin signaling is essential for lung development in utero, which has been elegantly investigated using the advantage of several wild-type and transgenic animals. Second, growing data suggest that WNT/β-catenin signaling is involved in pre- and postnatal lung injury and repair process; and third, several lines of evidence exists that highlight the impact of impaired WNT/β-catenin signaling on the development of adult chronic lung diseases, which seems similar to lung injury-repair processes in the developing lung. Additional studies are needed to advance our current knowledge of the pathogenesis of perinatal lung diseases, such as BPD, to shed further light into signaling pathways involved that ultimately might lead to novel therapeutic options for lung injury-repair process or epigenetic modifications in WNT signaling.
